# Understanding Relationships Between Chlamydial Infection, Symptoms, and Testing Behavior: An Analysis of Data from Natsal-3

**DOI:** 10.1097/EDE.0000000000001150

**Published:** 2020-02-03

**Authors:** Joanna Lewis, Peter J. White

**Affiliations:** From the a NIHR Health Protection Research Unit in Modelling Methodology and MRC Centre for Global Infectious Disease Analysis, Imperial College London School of Public Health, London, United Kingdom; b Modelling and Economics Unit, National Infection Service, Public Health England, London, United Kingdom.

**Keywords:** Chlamydia, Sexual risk behavior, Mass screening, Mathematical model, Statistical model, Bayesian analysis

## Abstract

Genital chlamydia is the most commonly diagnosed sexually transmitted infection worldwide and can have serious long-term sequelae. Numerous countries invest substantially in testing but evidence for programs’ effectiveness is inconclusive. It is important to understand the effects of testing programs in different groups of people.

**Methods:**

We analyzed data on sexual behavior and chlamydia tests from 16-to 24-year olds in Britain’s third National Survey of Sexual Attitudes and Lifestyles, considering test setting, reason, and result. We conducted descriptive analysis accounting for survey design and nonresponse, and Bayesian analysis using a mathematical model.

**Results:**

Most men testing due to symptoms tested in sexual health settings (63%; 95% confidence interval 43%–84%) but most women testing due to symptoms were tested by general practitioners (59%; 43%–76%). Within behavioral groups, positivity of chlamydia screens (tests not prompted by symptoms or partner notification) was similar to population prevalence. Screening rates were higher in women and in those reporting more partners: median (95% credible interval) rates per year in men were 0.30 (0.25–0.36) (0 new partners), 0.45 (0.37–0.54) (1 new partner), and 0.60 (0.50–0.73) (≥2 new partners). In women, they were 0.61 (0.53–0.69) (0 new partners), 0.89 (0.75–1.04) (1 new partner), and 1.2 (1.0–1.4) (≥2 new partners).

**Conclusions:**

Proportion of testing occurring in sexual health is not a proxy for proportion prompted by symptoms. Test positivity depends on a combination of force of infection and screening rate and does not simply reflect prevalence or behavioral risk. The analysis highlights the value of recording testing reason and behavioral characteristics to inform cost-effective control.

## Abstract 2

Supplemental Digital Content is available in the text.

Genital chlamydial infection is the most commonly diagnosed sexually transmitted infection (STI) worldwide, with an estimated 131 million new cases annually.^1^ Chlamydial infection in women is associated with increased risk of reproductive complications including pelvic inflammatory disease (PID), ectopic pregnancy, and tubal factor infertility.^2^

Widespread chlamydia testing is a major component of chlamydial control and sexual health policy in many high-income countries. There is moderate-quality evidence that chlamydia testing can reduce the risk of PID, but trials investigating its effect on prevalence have provided mixed conclusions.^3^ A recent review highlighted the need “to monitor population-based chlamydia incidence over time,”^4^ and indeed there is a wider need to understand the effects of large-scale testing programs on chlamydial infection in different groups of people. The World Health Organization has stated that “the best strategies to control and measure chlamydia infections are still to be defined.”^1^ As resources for public health become scarcer, it is important to develop surveillance systems and tools that can inform strategies for effective control and achieve optimal value for money.

England’s National Chlamydia Screening Programme (NCSP) opportunistically tests sexually active men and women <25 years of age. NCSP publishes valuable, near-complete data on the numbers of chlamydia tests and diagnoses in young people annually. Since the introduction of the NCSP in 2003 the annual number of tests per capita has increased, and the proportion of all tests that are positive (positivity) has fallen. We have recently developed a method^5^ that uses these data to estimate the prevalence of infection, taking into account the fact that testing is a mixture of tests not prompted by symptoms and diagnostic tests prompted by symptoms and conducted to determine their cause. These categories are not distinguished in the data, and so test setting is often used as a proxy, on the assumption that tests performed in sexual health settings are a mixture of the categories, while tests performed elsewhere are largely unprompted by symptoms.^6^

The third National Survey of Sexual Attitudes and Lifestyles (Natsal-3)^7^ took place after the completion of the national roll-out of the NCSP, and included both questions on chlamydia testing in the last year, and—in a subset of participants—urine sampling to test for chlamydial infection. Analysis by Sonnenberg et al^7^ and Woodhall et al^8^ found that some factors predicting prevalent chlamydial infection also predicted testing and diagnosis. A comparison between people tested by NCSP in 2008 and participants in the Natsal-2 survey (1999–2001) also showed that the former were more likely than the general population to be young, not to have used a condom at last sex, and to have had 2 or more partners in the last year: all factors also likely to predict infection. The NCSP data also showed that of those tested, people reporting nonuse of condoms and multiple partners were more likely to test positive.^9^

In this study, we use data from Natsal-3 to explore the mechanisms behind these findings. Because symptoms prompt testing, individuals with symptomatic infection are more likely to be tested than individuals with asymptomatic infection or no infection. We aim to discover whether this process alone explains the mutuality of factors associated with prevalent infection and with testing, or whether it is also the case that people without symptoms who are tested are at higher risk of infection than people who are not tested. We also examine the assumption that patients seeking diagnostic (i.e., symptomatic) testing are more likely to test in particular settings. Finally, we use our analysis to understand the population-level relationship between prevalence, testing rates, and positivity.

## METHODS

### Defining Sexual Risk

The term “risk behavior” is used in the literature in a number of subtly different ways. Here, we define the “risk” of an age, sex, behavioral, or other group in terms of the force of infection to which that group is exposed: the probability per person per unit time that an uninfected person in that group becomes infected. (In this article, “force of infection” in a group refers throughout to the force of infection acting on a group; not the force of infection it “exerts.”) A “high-risk” group has a high force of infection acting on it, while a “low-risk” group is exposed to a low force of infection. In general, this understanding of risk is aligned with “risk behavior”—i.e., a group will be exposed to a higher force of infection if individuals within the group have unprotected sex and/or have a large number of partners. However, if those partners are unlikely to be infected then the force of infection acting on the group will be low. The level of risk is influenced by both the behavior of the individuals in the particular risk group and the prevalence of infection among their partners. This definition of risk is in contrast to a definition in terms of prevalence within a group, which is influenced not only by force of infection but also by rates of diagnostic testing due to symptoms, testing unprompted by symptoms, and natural recovery.

### Data: Natsal-3

We used data from Natsal-3,^10^ a cross-sectional, stratified population-based survey of 15,162 men and women aged 16–74, conducted in Great Britain between 2010 and 2012. The response rate was 57.5%. Participants were interviewed using computer-assisted face-to-face and self-completion questionnaires. Urine samples were obtained for STI testing, with 1,832 of the 3,115 sexually experienced 16- to 24-year olds providing a useable sample.^8^ Findings from Natsal-3 regarding prevalence of and factors associated with chlamydial infection and with testing (e.g., age, sex, and behavioral characteristics) have been published elsewhere.^7,8^

Natsal-3 participants who reported having been tested for chlamydia in the last year were asked about the reason, setting, and result for their most recent test, and we analyze these data here. We classified tests according to the reported reason for testing, as (1) screens (“I wanted a general sexual health check-up”, “I had no symptoms but I was worried about the risk of Chlamydia,” or “I was offered a routine test”), (2) tests prompted by symptoms (“I had symptoms”), or (3) tests for any other reason. Location of test was classified as GP (“General practice (GP) surgery”) or SH (“Sexual health clinic (GUM clinic)”); all other locations, including internet tests, were classified as “other.”

### Descriptive Analysis

We examined the proportions of men and women in Natsal-3 16–24 years of age who had tested for chlamydia for different reasons and in different settings. We tested for associations between reason and setting using chi-squared tests. We grouped men and women by their reported number of new partners in the last year (0, 1, ≥2), and within each group we compared the positivity of all self-reported tests in the last year, all tests *except* positive tests prompted by symptoms, and all tests defined as screens (using the definition of “screen” above). We conducted descriptive analyses in the R environment^11^ using the *survey*^12^ package, and accounted for survey design and nonresponse using stratification variables and nonresponse weights.

### Model-based Analysis

We also analyzed the data using a mathematical model of chlamydial infection, testing, and recovery, which is described fully in online supplemental material (eAppendix 1; http://links.lww.com/EDE/B625). Briefly, the model classifies incident infections as either symptomatic or asymptomatic. All infections can clear naturally or be detected through testing, and symptomatic infections prompt diagnostic testing due to active care-seeking. Model inputs are the force of infection, natural history parameters (proportion of incident infections that are symptomatic and clearance rate of untreated infections), and rates of screening and active treatment-seeking. The outputs are observed numbers of tests in patients with and without symptoms, and positive and negative test results. In the model-based analysis, tests are classified as symptomatic diagnoses, versus all other tests—including those prompted by partner notification, partner’s symptoms, a previous positive test, or “other” reasons. In eAppendix 1; http://links.lww.com/EDE/B625, we present a structural sensitivity analysis using an extended model in which testing due to partner notification is distinguished from other testing not prompted by symptoms.

We used a Bayesian framework to infer force of infection and screening rate in our model. The statistical model is described fully in eAppendix 1; http://links.lww.com/EDE/B625. We used informative prior distributions for the natural history parameters and treatment-seeking rate. We used uninformative priors for the force of infection and screening rate. We calculated the likelihood of the data for each Natsal-3 respondent according to whether they reported testing for chlamydia in the last year and, if they had been tested, the test result and whether they reported that the test was prompted by symptoms. We weighted individual log-likelihoods in the full log-likelihood using the survey weights. As a validation check, we compared prevalence inferred by the model with reported prevalence estimates.^8^

We conducted separate analyses for men and women, with and without stratification by the reported number of new partners in the last year. In the unstratified analysis, all parameters were the same for all respondents of a particular sex. In the stratified analysis, we allowed force of infection and screening rate to differ between behavioral groups (0, 1, ≥2 new partners in the last year), but natural history parameters and the treatment-seeking rate in response to symptoms were the same.

We inferred posterior parameter distributions by Markov Chain Monte Carlo sampling using the Stan software,^13^ in the R environment. We generated 1000 warmup samples, followed 9000 posterior samples. All code used for analysis is available online at https://github.com/joanna-lewis/natsal3_ct_testing.

### Ethics

This study used only archived, publicly available data from the Natsal-3 study. The Natsal-3 study was approved by Oxfordshire Research Ethics Committee A (reference number: 10/H0604/27).

## RESULTS

### Descriptive Analysis: Is Test Setting Associated with Reason for Test?

**FIGURE 1.**
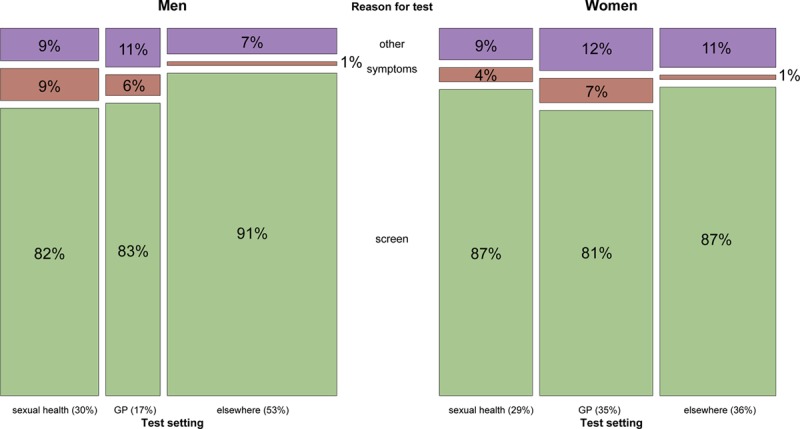
Reasons for and settings of most recent chlamydia test within the last year, for 16- to 24-year-old men and women responding to Natsal-3. In each plot, the width of the columns indicates the percentage of all tests that occurred in each setting. Percentages are given below the columns. Each vertical column is split and labeled to indicate the percentage of tests in a setting that were for each reason: screens (green), prompted by symptoms (brown), or occurred for other reasons (purple).

Overall, 35% (95% confidence interval: 32%–37%) of men and 54% (51%–57%) of women aged 16–24 and reporting at least 1 partner ever, reported testing for chlamydia in the last year.^7^ Figure 1 illustrates the distribution of these tests by reported reason and setting. For men, the largest proportion of reported tests were outside GP or sexual health services (53%; 48%–58%), with only 17% (13%–21%) of tests in GPs and 30% (26%–35%) in sexual health. For women similar proportions were in general practice (35%; 32%–38%) and “elsewhere” (36%; 33%–39%), with only 29% (25%–33%) of tests in sexual health. Most tests were screens (87%; 84%–90% in men, and 85%; 82%–88% in women). Positivity was much higher in sexual health than in other settings (15%; 9%–23% vs 2%; 1%–4% in men and 10%; 7%–14% vs 4%; 2%–6% in women).

The association between test setting and reason for test differed between the sexes. Most men testing due to symptoms used sexual health (63%; 43%–84%), and there was strong evidence of an association between testing due to symptoms and testing in sexual health (chi-squared test for survey data; *P* = 0.0010). Of all tests in men in sexual health, 9% (5%–16%) were prompted by symptoms, compared with 2% (1%–4)% in other settings (Table 1). In contrast, most women who reported that symptoms prompted their last test were tested by GPs (59%; 43%–76%). In women, there was no evidence of an association between testing in sexual health and testing due to symptoms (*P* = 0.9992), with 4% of women’s testing both within (95% CI 2%–8%) and without (95% CI 3%–6%) sexual health clinics reported to be in response to symptoms.

**TABLE 1.**
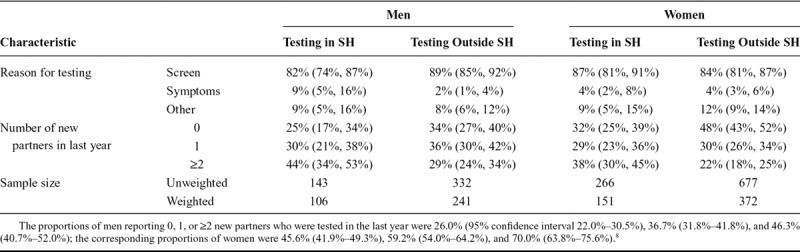
Characteristics of Sexually Experienced 16- to 24-Year-Old Men and Women Testing in Sexual Health Services (SH) and Elsewhere

In both men and women, those testing in sexual health tended to have more partners than those testing in other settings, although the evidence was stronger in women (chi-squared test for survey data; *P* < 0.0001) than in men (*P* = 0.028). Therefore, in women there is evidence that the higher positivity in sexual health than other settings is associated with higher numbers of partners, but no evidence that it is associated with testing due to symptoms. In contrast, the higher positivity in men testing in sexual health compared with testing elsewhere is associated both with having more partners and with a tendency among symptomatic men to test in sexual health.

### Descriptive Analysis: How Do Test Positivity and Prevalence of Chlamydial Infection Compare in People Reporting Different Sexual Behavior, Testing for Different Reasons?

**FIGURE 2.**
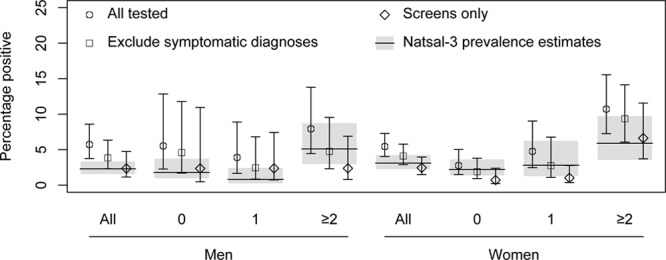
Positivity of reported tests in men and women reporting different numbers of new partners in the last year, and testing for different reasons. Markers and error bars show estimated (95% confidence interval) positivity of tests reported by all men and women, and stratified into those reporting 0, 1, or ≥2 new partners in the last year. The estimates marked by circles include all tests; squares exclude diagnoses from testing due to symptoms, and diamonds include only tests classified as screens (see Methods). The gray bars show the population prevalence in the same groups (estimate and 95% confidence interval), from Woodhall et al.^8^

Figure 2 shows the self-reported positivity of tests in the last year reported in Natsal-3 by respondents with different reasons for testing, with stratification into risk behavior categories. The positivity of all tests (left-hand bar of each triplet) corresponds to surveillance data from the NCSP, which reports on all tests without differentiating by reason. Excluding diagnoses prompted by symptoms (middle bar of each triplet) corresponds to the testing not prompted by symptoms in our model-based analysis. As expected, the point-estimate positivity of these tests is lower than that of all tests. Including only tests specifically identified as screens excludes all reasons for testing that might be predictive of infection: for example, partner notification or a previous positive result. In each sex overall, and in almost every risk category, the point-estimate positivity of screens (right-hand bar of each triplet) was similar to, or even lower than, the population prevalence for that group as estimated from the Natsal-3 urine samples^8^—indicating that infected people are no more likely than uninfected people be screened.

We conducted an additional analysis, in which the “middle” positivity estimate excluded not only symptomatic diagnoses but also testing due to partner notification. The results were similar (eFigure 1; http://links.lww.com/EDE/B625).

### Model-based Analysis: Did Screening Rate Vary Between Risk Groups in Natsal-3?

**FIGURE 3.**
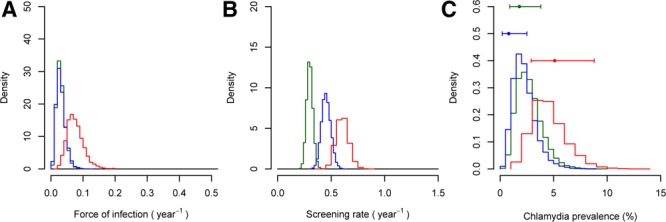
Evidence synthesis to infer force of infection, screening rate and prevalence of chlamydial infection in women 16–24 years of age. The 3 panels show posterior distributions for (A) force of infection, (B) screening rate, and (C) prevalence. Green, blue, and red indicate results from women reporting 0, 1, or ≥2 new partners in the last year, respectively. The points and error bars in panel C indicate observed prevalence, with the 95% confidence interval, from Woodhall et al.^8^

**FIGURE 4.**
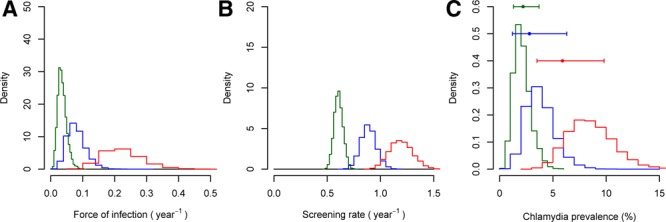
Evidence synthesis to infer force of infection, screening rate, and prevalence of chlamydial infection in men 16–24 years of age. The 3 panels show posterior distributions for (A) force of infection, (B) screening rate, and (C) prevalence. Green, blue, and red indicate results from women reporting 0, 1, or ≥2 new partners in the last year, respectively. The points and error bars in panel C indicate observed prevalence, with the 95% confidence interval, from Woodhall et al.^8^

Our model-based analysis explains how force of infection and screening rate can jointly affect the prevalence of chlamydial infection. In women (Figure 3; Table 2), we found higher force of infection and higher screening rates in those who reported more new partners in the last year. Median (95% credible interval) force of infection was 0.034 (0.014, 0.068) per year in women reporting 0 new partners, 0.077 (0.035, 0.148) in women reporting 1, and 0.22 (0.12, 0.36) in women reporting ≥2. Screening rate was 0.61 (0.53–0.69) per year in women reporting 0 new partners, 0.89 (0.75–1.04) in women reporting 1, and 1.2 (1.0–1.4) in women reporting ≥2. However, the higher screening rates were not high enough to counter the greater force of infection so prevalence was higher in those with more partners. In contrast, in men (Figure 4; Table 3) the force of infection was similar in those reporting 1 (0.029 [0.010–0.064] per year) versus 0 (0.029 [0.012–0.061]) new partners, but screening rate was higher (0.45 [0.37–0.54] per year vs. 0.30 [0.25–0.36]), causing the model to predict a lower prevalence: a prediction which agrees with the observed prevalence. Men reporting ≥2 new partners in the last year had the highest screening rate (0.60 [0.50–0.73]), but also had a force of infection much higher than the other groups (0.073 per year [0.035–0.136]), resulting in a higher prevalence. In all risk groups, there was close agreement between prevalence inferred by our model and direct estimates previously reported,^8^ even though the model was not fitted directly to the prevalence estimates. In summary, the model both estimates prevalence correctly and also explains the infection dynamics underlying it. We note that the posterior distribution for prevalence inferred in the unstratified analysis also agreed well with the observed unstratified prevalence (Tables 2 and 3, eFigures 6 and 7; http://links.lww.com/EDE/B625), that is, the unstratified model infers overall prevalence accurately, despite the heterogeneity in screening rates and force of infection.

**TABLE 2.**
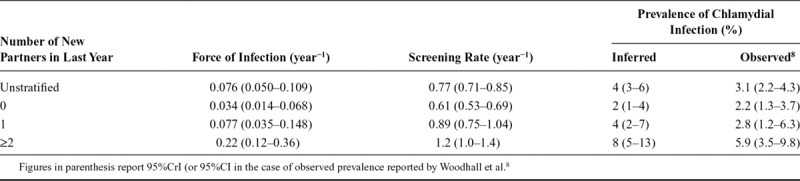
Posterior Distributions for Evidence Synthesis in Women

**TABLE 3.**
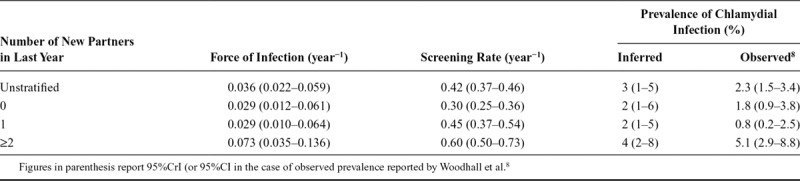
Posterior Distributions for Evidence Synthesis in Men

The structural sensitivity analysis produced similar results (see online supplemental material; http://links.lww.com/EDE/B625).

### Model-based Analysis: How Do Force of Infection and Screening Rate Affect Positivity?

**FIGURE 5.**
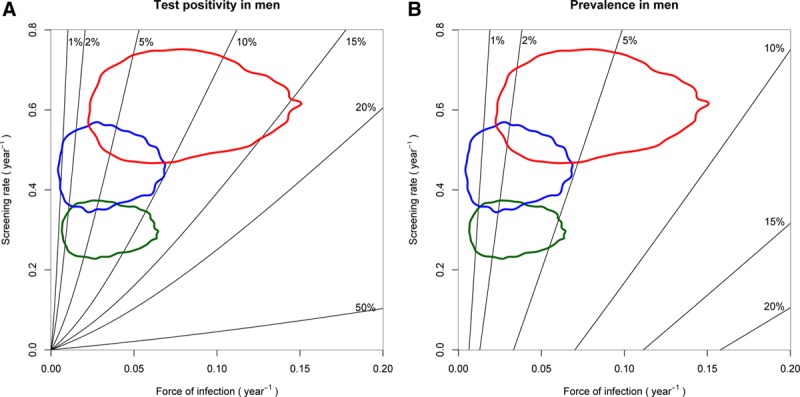
Test positivity (A) and prevalence of chlamydial infection (B) predicted by our model in men exposed to differing forces of infection and screening rates. Black contours indicate positivity (A) and prevalence (B). Colored contours show the force of infection and screening rate for each risk group, each enclosing 95% of the (force of infection, screening rate) samples for people in that group. Green, blue, and red contours correspond to people reporting 0, 1, or ≥2 new partners in the last year. Equivalent figures for women are provided in online supplemental material (eFigure 5; http://links.lww.com/EDE/B625.

Having found that force of infection and screening rate differ between behavior groups, we used our model to predict how these two quantities would affect observed positivity. In Figure 5A, black contours show how overall test positivity varies with force of infection and screening rate in men. Figure 5B shows how prevalence varies over the same range. (Equivalent plots for women are included in eFigure5; http://links.lww.com/EDE/B625.) Importantly, positivity is determined not only by risk (force of infection), but also by the screening rate. If force of infection is low, and/or screening rate is high, then positivity is low because the prevalence of infection is low, there are few symptomatic infections, and most tests are screens. If the force of infection is higher, and/or screening rate is lower, then a greater proportion of tests are a result of incident symptomatic infection and population prevalence is higher, so the positivity of those tested is higher.

If the screening rate increases then positivity will fall even if the force of infection remains the same. The colored contours shown on Figure 5 illustrate this point by showing the distribution of force of infection and screening rate inferred by the model for men reporting 0, 1, and ≥2 new partners. For example, the green and blue contours in Figure 5A show that men reporting 0 and 1 new partners in the last year had similar force of infection, but men in the latter group had a higher screening rate, which led to a lower positivity. Positivity is greatest in men reporting ≥2 new partners because although their screening rate is high compared with those reporting fewer partners, this effect is outweighed by their high force of infection.

## DISCUSSION

We analyzed chlamydia testing and diagnosis patterns using data from the population-based Natsal-3 survey and a combination of descriptive statistics and model-based inference techniques, validated by comparing inferred prevalence with published estimates.^8^ We found that men reporting testing due to symptoms were most likely to be tested in sexual health clinics, but women with symptoms were most likely to be tested by GPs. The proportion of tests that were carried out in sexual health is therefore not a reliable indicator of the proportion of tests that were prompted by symptoms, as is often assumed (e.g., Public Health England^6^).

Mercer et al.^14^ investigated the relationship between test setting and reason for test in men and women combined, in sexual health and GP-based “Locally Enhanced Services” in a county in England, using questionnaires linked to clinical records. Although the study found that patients in sexual health were more likely than GP patients to have symptoms at the time of consultation, there was no difference in the proportion of patients reporting symptoms as the *reason* for seeking care. This is consistent with our finding, based on a nationally representative survey, that the proportion of chlamydia tests that were requested in sexual health is not a proxy for the proportion of tests prompted by symptoms.

Our results add to previous analysis of Natsal-3 data by Sonnenberg et al,^7^ and Woodhall et al.^8^ Prior to our analysis, it was not known whether the association between chlamydial infection and testing could be explained simply by the mediating effect of symptomatic infection in prompting testing. We addressed this by using data on the reason for testing in descriptive and model-based analysis. We found that, as expected, positivity varied by reason for testing, with the point-estimate positivity of all reported chlamydia tests (including those prompted by symptoms) being higher than the positivity of tests not prompted by symptoms, which was in turn higher than the positivity of tests identified as a “general check-up,” “routine test” or prompted by being “worried about the risk of chlamydia”—although small numbers of positives meant the confidence intervals were wide. The positivity in the final category was similar to the population prevalence in both men and women overall, and no higher than population prevalence in all but one behavior-stratified group.

Our model-based analysis allowed us to understand patterns of prevalence and test positivity in men and women reporting different sexual behavior. By including the additional data on reason for test recorded in Natsal-3 in our model-based analysis, we have found that the higher testing rate in people reporting more new partners is partly a result of higher incidence of infection leading to higher incidence of symptoms prompting testing, but that there is also a higher rate of screening than in people with fewer partners. As a group, women reporting more partners were exposed to a higher force of infection and had a higher screening rate, and because the screening rate was not high enough to counteract the higher force of infection, the prevalence was also elevated (Figure 3; Table 2). Men reporting 0 and those reporting 1 new partner were exposed to a similar force of infection, but a higher screening rate in those with one new partner led to a lower prevalence (Figure 4; Table 3). We also note that an unstratified model was able to infer prevalence accurately, in spite of population heterogeneities in risk and screening rate. Finally, our analysis illustrates how positivity is determined by a combination of force of infection (risk) and screening rate (Figure 5). In England, chlamydia testing rates increased and positivity fell every year from 2000 to 2010,^15^ which has been interpreted as a decreasing average sexual behavior risk profile of those tested as testing increased.^16^ Our analysis shows that a fall in positivity could be explained by a reduced force of infection *or* an increased screening rate—or a combination of the two—but it does not necessarily require a changing bias in screening towards those who have lower-risk behavior.

A major strength of our study is the use of data from the population-based Natsal-3 survey. Information was available on reason for test as well as test setting and test result, and it was possible to adjust for nonresponse using the survey weights. Limitations are that information was self-reported, based on recall, and that the Natsal-3 questionnaire did not provide guidance about the symptoms of chlamydial infection. Also, as symptoms are nonspecific it is possible that respondents could have sought testing in response to symptoms that were not linked to chlamydial infection; however, the incidence of symptomatic infection with other sexually transmitted infections was very low.^7,17^ Interestingly, the posterior distribution for the proportion of infections that are symptomatic in men suggests a lower proportion than the prior. The prior is based on a small study, with a rather broad definition of symptoms (any urethral discharge). The Natsal-3 data adds information to the prior and suggests a lower proportion of infections symptomatic—more in line with figures that have been used in modeling studies. Finally, small numbers of infections mean that there is wide uncertainty in estimates from Natsal-3 (Figures 3 and 4) and that, like others,^8,9^ we had to combine categories of response to the questions about reason and setting.

Our study, based on detailed data from Natsal-3, highlights the importance of a careful interpretation of surveillance data from chlamydia screening and the value that collecting additional data on sexual behavior and reason for test can add. Current surveillance in England provides some of the best data available on a national-level chlamydia testing program, which can be used to estimate the incidence and prevalence of infection at a national and local level.^5,18^ However, if we are to rigorously evaluate screening programs and optimize them, including targeted testing of those most likely to be infected, then more-detailed data are required. We have gained insights using data from 1418 people 16–24 years of age who reported chlamydia testing in the last year in the nationally representative Natsal-3 survey. NCSP conducted over 1.3 million tests in 15- to 24-year olds in 2018^6^ and could collect a very powerful data set that could be analyzed using the techniques presented here to investigate the infection and testing dynamics underlying patterns of prevalence in different demographic and behavioral groups and different local authorities, and to understand the reasons for the inequalities across local authorities.^5^ A chlamydia testing dataset including the data used in our analysis: reason for testing (particularly presence/absence of symptoms^5^), and behavioral factors, and detailed, accurate geographical information (e.g., postcode) would better inform cost-effective targeting of services to provide for those in greatest need.^5^

## Supplementary Material


