# Supplementary material for: Understanding Relationships Between Chlamydial Infection, Symptoms, and Testing Behavior: An Analysis of Data from Natsal-3
Source: Epidemiology. 2020 Feb 3;31(2):263–71. doi: 10.1097/EDE.0000000000001150 (PMC7004460; doi:10.1097/EDE.0000000000001150)
Supplement: Supplementary file 1 [file ede-31-263-s001.pdf]

Understanding relationships between chlamydia infection, symptoms and testing behavior: an analysis of data from Natsal-3

Joanna Lewis and Peter J White

Online Supplemental Material: eAppendix 1

Contents

1. Descriptive analysis: additional information
    1.1 Classification of reason for testing
    1.2 Positivity: supplementary results
2. Model-based analysis: main model specification
    2.1 Mathematical model
    2.2 Statistical model
        2.2.1 Priors
        2.2.2 Likelihood
    2.3 Model-based analysis: Additional results
        2.3.1 Did screening rate vary between risk groups in Natsal-3?
        2.3.2 How do force of infection and screening rate affect positivity?
3. Model structural sensitivity analysis: unstratified analysis
4. Model structural sensitivity analysis: partner notification
    4.1 Mathematical model
    4.2 Statistical model
    4.3 Results
5. References

1. Descriptive analysis: additional information

1.1. Classification of reason for testing

Natsal-3 participants who reported having been tested for chlamydia in the last year were asked about the reason for their most recent test. Each respondent could choose one of eight possible answers, given in the left-hand column of eTable 1. In the main analysis (see main text) we compared the positivity of all tests, tests *excluding* those that were prompted by symptoms and had a positive result, and known screens. In the supplementary analysis (see below) we compared all tests, tests *excluding* those that were prompted by symptoms and had a positive result and *excluding* partner notifications, and known screens. eTable 1 shows how reported reasons for testing were classified in these comparisons.

eTable 1: Classification of reason for test in the positivity comparisons reported in the main text and online supplemental material.

**Reported reason for testing**	**Number reporting each reason** (unweighted)		All tests	Excluding symptomatic diagnoses (main analysis)	Excluding symptomatic diagnoses and partner notifications (supplemental material)	Known screens only
	Men	Women				
I had symptoms (positive result)	8	14	✓			
I was notified because a partner was diagnosed with Chlamydia	4	15	✓	✓		
I had symptoms (negative result)	14	27	✓	✓	✓	
Check up after previous positive test	4	10	✓	✓	✓	
My partner had symptoms	15	11	✓	✓	✓	
Other	20	71	✓	✓	✓	
I wanted a general sexual health check-up	198	363	✓	✓	✓	✓
I had no symptoms but I was worried about the risk of Chlamydia	50	77	✓	✓	✓	✓
I was offered a routine test	162	355	✓	✓	✓	✓

1.2. Positivity: supplementary results

We compared the positivity of tests carried out for different reasons using a similar analysis to that described in the main text, but excluding tests due to notifications by diagnosed partners as well as symptomatic diagnoses from the "middle" positivity estimate. The results are shown in eFigure 1. Results were similar to the main analysis. In most risk groups the positivity excluding both notifications and symptomatic diagnoses was slightly lower than excluding symptomatic diagnoses only, but small numbers of partner notifications meant the difference was small, and the confidence intervals are wide.

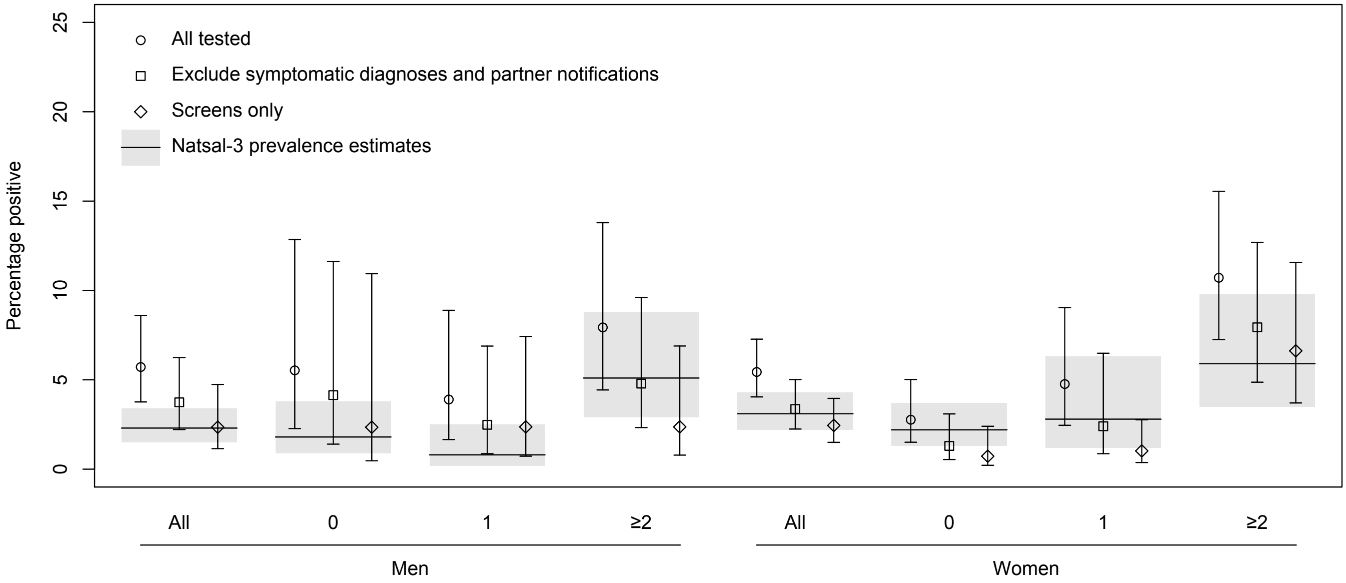

eFigure 1: Positivity of reported tests in men and women reporting different numbers of new partners in the last year, and testing for different reasons. *Markers and error bars show estimated (95% CI) positivity of tests reported by all men and women and those reporting 0, 1 and ≥2 new partners. The estimates marked by circles include all tests; squares exclude notifications by infected partners and diagnoses reported as prompted by symptoms, and diamonds include only tests classified as screens (see methods). The gray bars show the population prevalence in the same groups (estimate and 95% CI).[1]*

2. Model-based analysis: main model specification

2.1. Mathematical model

eFigure 2 illustrates the mathematical model used in our analysis, which divides population of interest into three compartments: uninfected (*U*), infected-symptomatic (*S*) and infected-asymptomatic (*A*). Most of the population are uninfected, and can become infected with a constant force of infection (*foi*). A proportion (p_{symp}) of incident infections develop symptoms, and individuals move to the infected-symptomatic compartment. The remainder ($1 - p_{symp}$) become asymptomatic infections. All infections can be cleared naturally by the immune system, at rate λ, or may be diagnosed and treated through a screening program, at rate *scr*. In addition, symptomatic infections cause individuals to seek testing and treatment, at rate *trt*. Natural clearance, screening and treatment-seeking all return infected individuals to the uninfected compartment.

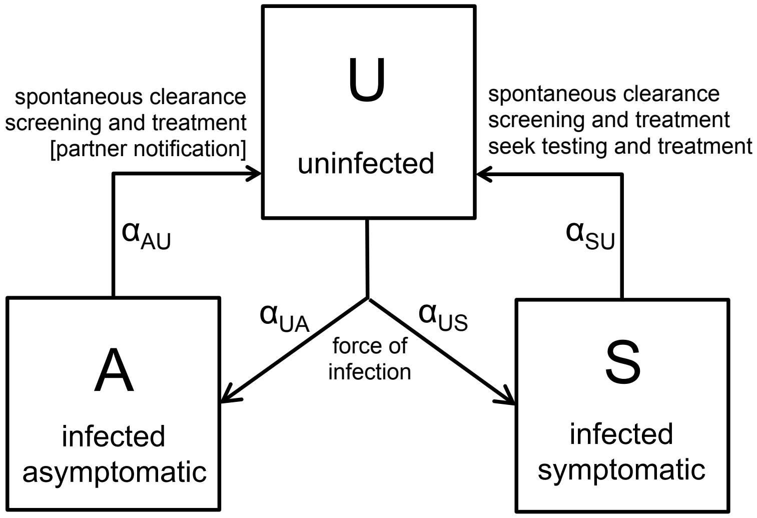

***eFigure 2: Mathematical model used in the model-based analysis** showing the processes by which Individuals move between uninfected (U), infected-asymptomatic (A) and infected-symptomatic (S) states.*

The model can be represented by differential equations as follows:

$$\frac{dU}{dt} = \alpha_{AU}A + \alpha_{SU}S - (\alpha_{UA} + \alpha_{US})U$$

$$\frac{dA}{dt} = \alpha_{UA}U - \alpha_{AU}A$$

$$\frac{dS}{dt} = \alpha_{US}U - \alpha_{SU}S$$

Where:

$$\begin{aligned} \alpha_{AU} &= \lambda + scr \\ \alpha_{SU} &= \lambda + scr + trt \\ \alpha_{UA} &= foi\left(1 - p_{symp}\right) \\ \alpha_{US} &= foi\, p_{symp} \end{aligned}$$

At steady state, when all derivatives are equal to zero, the equations can be solved to give the proportion of the population in each compartment:

$$U = \frac{\alpha_{SU}\alpha_{AU}}{\alpha_{AU}\alpha_{US} + \alpha_{SU}(\alpha_{AU} + \alpha_{UA})}$$

$$A = \frac{\alpha_{SU}\alpha_{UA}}{\alpha_{AU}\alpha_{US} + \alpha_{SU}(\alpha_{AU} + \alpha_{UA})}$$

$$S = \frac{\alpha_{AU}\alpha_{US}}{\alpha_{AU}\alpha_{US} + \alpha_{SU}(\alpha_{AU} + \alpha_{UA})}$$

The prevalence of infection is the sum of the proportion of infections in the asymptomatic-infected and symptomatic-infected compartments:

$$\text{prevalence} = A + S = \frac{\alpha_{SU}\alpha_{UA} + \alpha_{AU}\alpha_{US}}{\alpha_{AU}\alpha_{US} + \alpha_{SU}(\alpha_{AU} + \alpha_{UA})}$$

2.2. Statistical model

We conducted Bayesian inference by MCMC sampling, based on our model and using the Natsal-3 data on chlamydia testing and diagnosis.

2.2.1. Priors

The proportion of incident infections that become symptomatic (i.e. enter compartment S) was informed by a study of men and women returning to an STD clinic for treatment following a positive chlamydia test.[2] Ten of 14 men and 26 of 115 women returning had urethral/cervical discharge. We therefore used a Beta(11, 5) prior for p_{symp} in men and a Beta(27, 90) prior in women. Priors for the natural chlamydia clearance rate were based on the results of previous evidence syntheses;[3,4] we used lognormal(log(0.42), 0.4) in men and normal(0.74, 0.071) in women.

We used a gamma(14,1) prior for the rate of treatment seeking by symptomatic individuals, *trt*, based on previous analysis[5] of data from a sexual health clinic.[6] We used uninformative priors (exponential(0.001)) for the force of infection *foi* and screening rate *scr.*

2.2.2. Likelihood

The full log-likelihood was calculated by summing the log-likelihood of each individual's testing data, multiplied by their survey weight.

- For individuals who were not tested for chlamydia in the last year, the likelihood was:
$$P_{Poisson}(0|scr + S\ trt)$$

- For individuals who were diagnosed with chlamydia in the last year in response to symptoms the likelihood was the product (i.e. the log-likelihood was the sum) of two components:
  1. The likelihood associated with testing (as opposed to not testing):
  $$1 - P_{Poisson}(0|scr + S\ trt)$$
  2. The probability that a test chosen at random from all the tests reported was prompted by symptoms:
  $$\frac{S\ trt}{scr + S\ trt}$$

- For individuals who were tested for chlamydia in the last year but not in response to symptoms, or were tested in response to symptoms but found to be uninfected, the likelihood was the product (i.e. the log-likelihood was the sum) of three components:

  1. The likelihood associated with testing (as opposed to not testing):
  $$1 - P_{Poisson}(0|scr + S\ trt)$$
  2. The probability that a test chosen at random from all the tests reported was not one prompted by symptoms:
  $$\frac{scr}{scr + S\ trt}$$
  3. The likelihood of the test result:
  $$\text{prevalence} = A + S \quad \text{if test positive}$$
  $$1 - \text{prevalence} = U \quad \text{if test is negative}$$

In the model, screening samples individuals randomly from the population, so the probability that a screen is positive equals the population prevalence.

2.3. Model-based analysis: Additional Results

2.3.1. Did screening rate vary between risk groups in Natsal-3?

eFigures 3 and 4 show prior and posterior distributions for all five model parameters: proportion of infections symptomatic, symptomatic treatment rate, natural clearance rate, force of infection and screening rate. The final panel in each figure also shows the posterior distribution for prevalence.

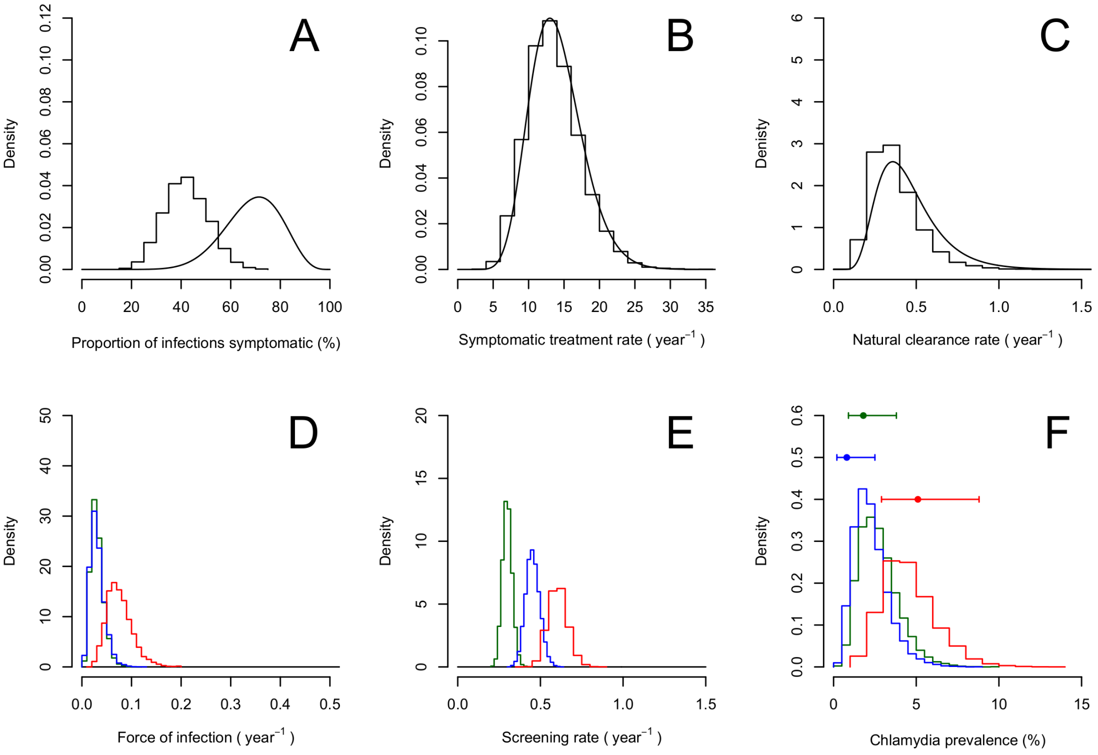

eFigure 3: Evidence synthesis to infer force of infection, screening rate and prevalence of chlamydial infection in men aged 16-24. *Panels A-E each show prior (smooth curve) and posterior (histogram) distributions for one parameter of the model. (The uninformative priors in panels D and E appear as horizontal lines on this scale.) Panel F shows posterior distributions for prevalence. Where more than one histogram is shown, green, blue and red indicate results from men reporting 0, 1 or ≥2 new partners in the last year, respectively. The points and error bars in panel F indicate observed prevalence, with the 95% confidence interval, from [7].*

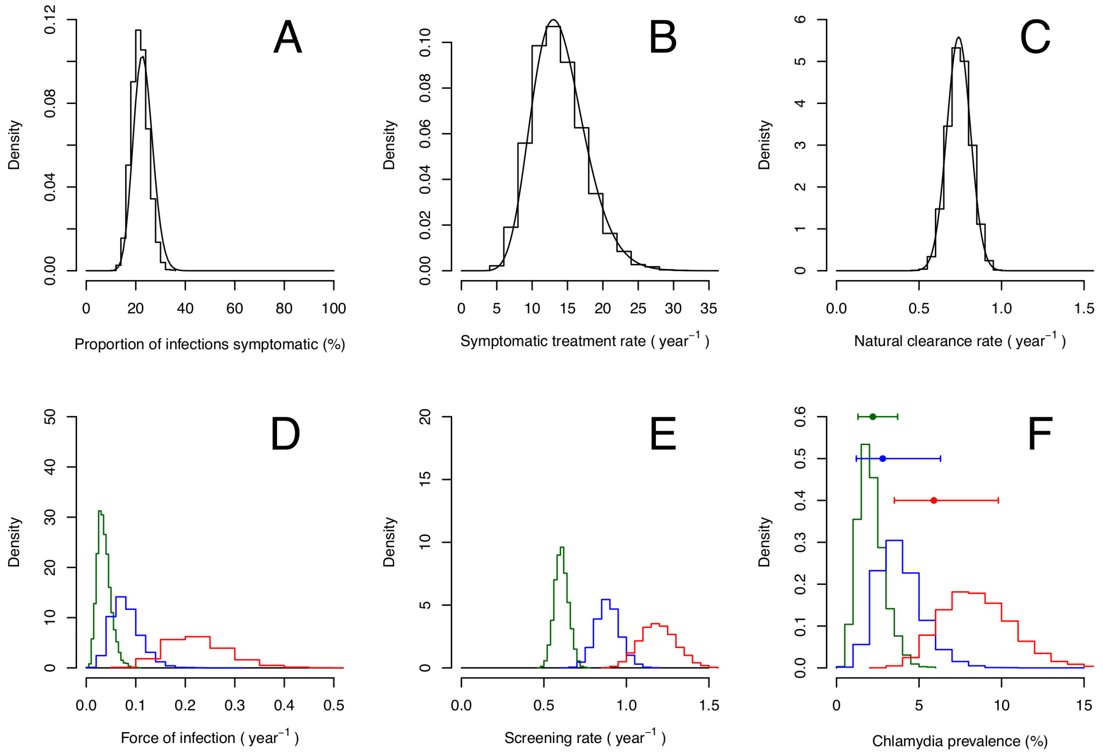

eFigure 4: Evidence synthesis to infer force of infection, screening rate and prevalence of chlamydial infection in women aged 16-24. *Panels A-E each show prior (smooth curve) and posterior (histogram) distributions for one parameter of the model. (The uninformative priors in panels D and E appear as horizontal lines on this scale.) Panel F shows posterior distributions for prevalence. Where more than one histogram is shown, green, blue and red indicate results from women reporting 0, 1 or ≥2 new partners in the last year, respectively. The points and error bars in panel F indicate observed prevalence, with the 95% confidence interval, from [7].*

2.3.2. How do force of infection and screening rate affect positivity?

eFigure 5 shows the same material as Figure 5 in the main text, alongside equivalent panels for women.

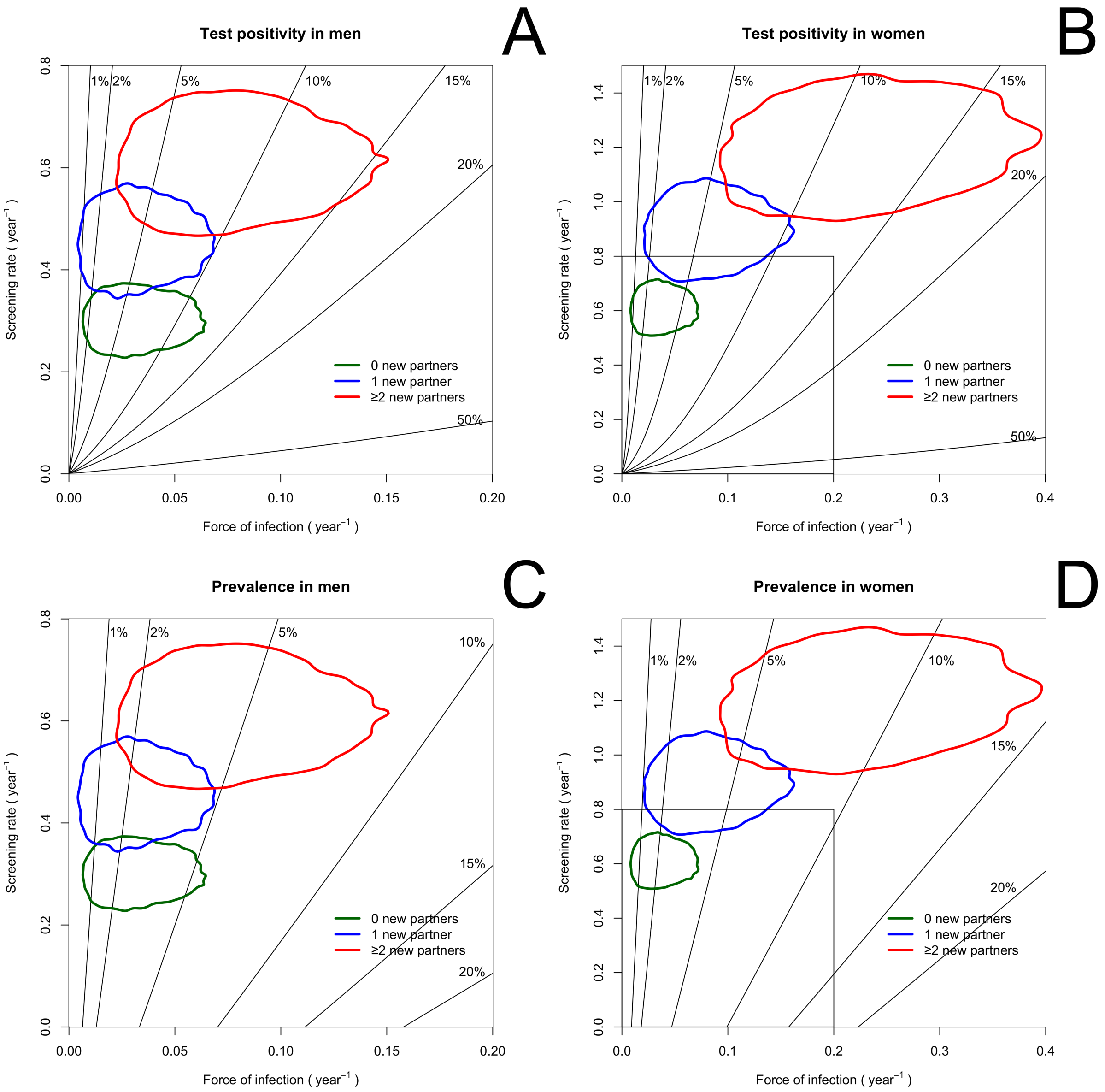

eFigure 5: Test positivity (A,B) and prevalence of chlamydial infection (C,D) predicted by our model in (A,C) men and (B,D) women exposed to differing forces of infection and screening rates. *Black contours indicate positivity (A,B) and prevalence (C,D). Colored contours show the force of infection and screening rate for each risk group, each enclosing 95% of the (force of infection, screening rate) samples for people in that group. Green, blue and red contours correspond to people reporting 0, 1 and ≥2 new partners in the last year. Note that the scales in the two panels are different: the black box in panels B and D (women) shows the extent of the axes in panels A and C (men).*

3. Model structural sensitivity analysis: unstratified analysis

In the main text we present the model-based analysis with men and women stratified by reported number of new partners in the last year. eFigures 6 and 7 show the results of analysis without this stratification. The posterior distributions for the proportion of incident infections that are symptomatic, the symptomatic treatment rate, and the natural clearance rate, are very similar to those in the stratified analysis (main text). The posterior distributions for force of infection and screening rate are intermediate within the range of the three posterior distributions in the stratified analysis. Notably, the posterior distributions for chlamydia prevalence in both men and women agree well with the corresponding prevalence estimates observed in Natsal-3.[7]

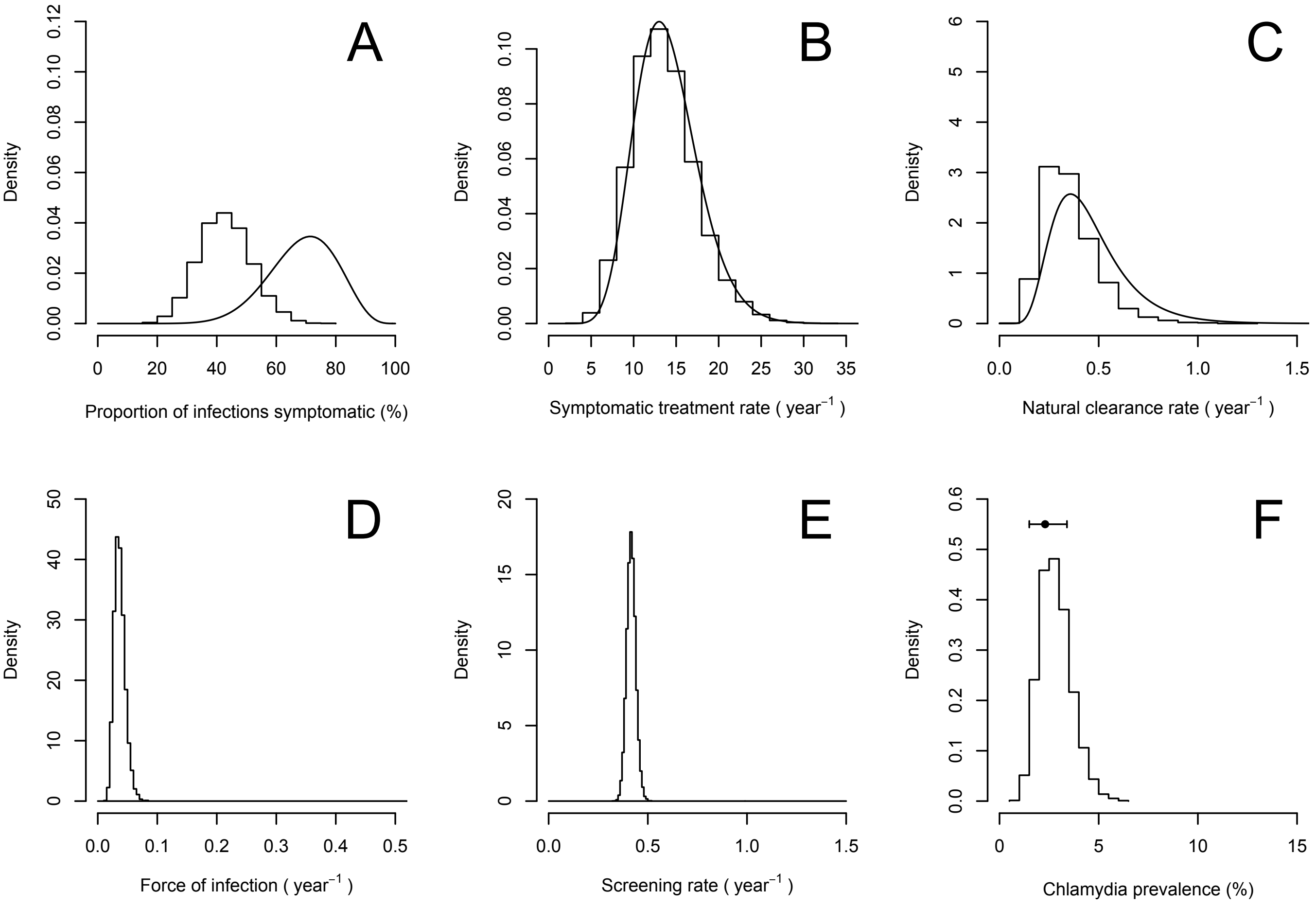

eFigure 6:* *Evidence synthesis to infer unstratified force of infection, screening rate and chlamydia prevalence in men aged 16-24. *Panels A-E each show prior (smooth curve) and posterior (histogram) distributions for one parameter of the model. Panel F shows the posterior distribution for prevalence. The marker and error bar in panel F indicates observed prevalence reported in [1] with the 95% confidence interval.*

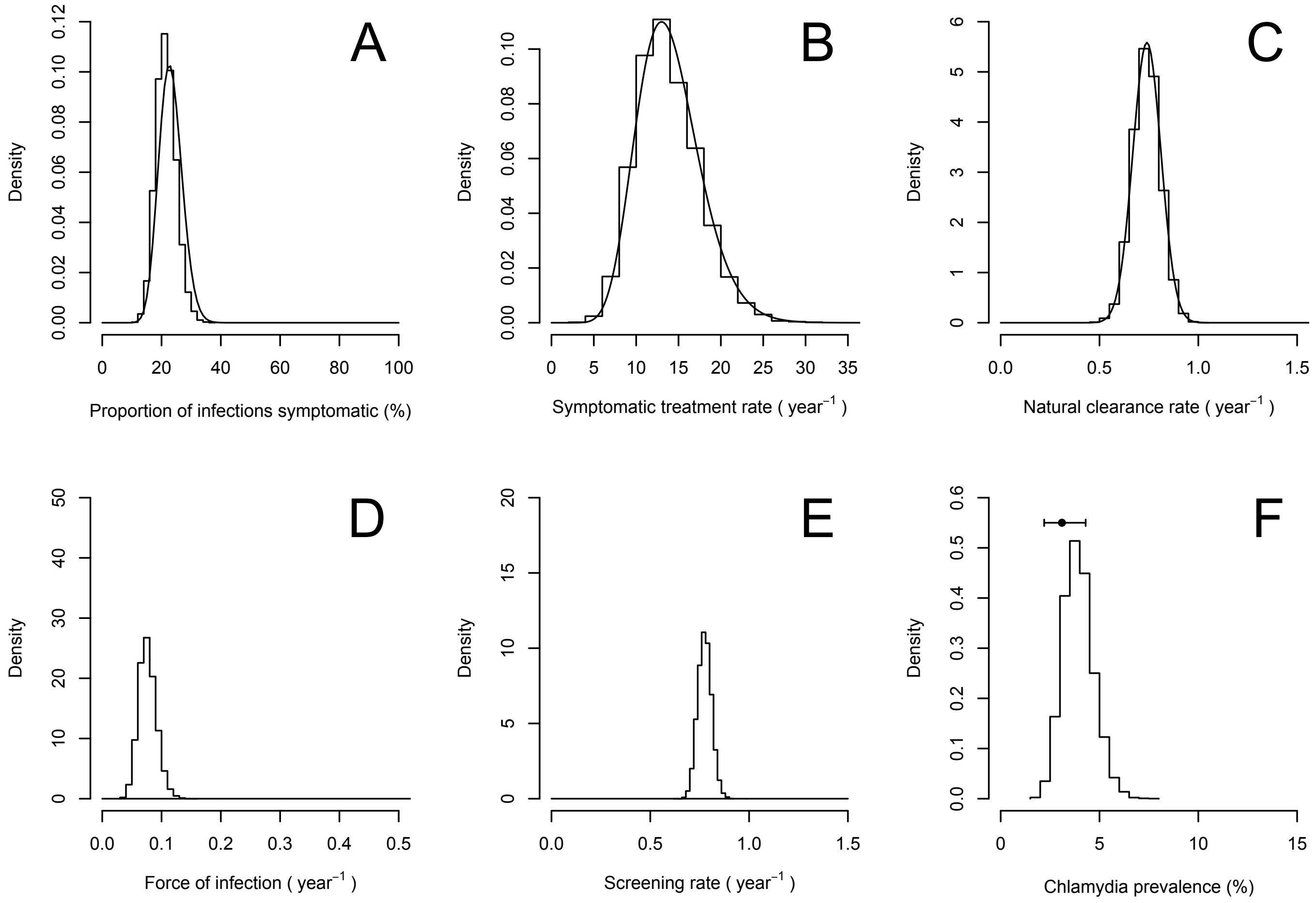

eFigure 7:* *Evidence synthesis to infer unstratified force of infection, screening rate and chlamydia prevalence in women aged 16-24. *Panels A-E each show prior (smooth curve) and posterior (histogram) distributions for one parameter of the model. Panel F shows the posterior distribution for prevalence. The marker and error bar in panel F indicates observed prevalence reported in [1] with the 95% confidence interval.*

4. Model structural sensitivity analysis: partner notification

We extended our model of chlamydia infection, testing and diagnosis to distinguish testing in response to partner notification from other testing not prompted by symptoms.

4.1. Mathematical model

The mathematical model incorporating partner notification is identical to the original model, except that it includes two additional parameters, pn_U and pn_A, representing testing by uninfected individuals and infected-asymptomatic individuals respectively, in response to partner notification. Partner notification affects the dynamics of the mathematical model through the rate at which asymptomatic-infected people are tested and treated: in the model with partner notification,

$$\alpha_{AU} = \lambda + scr + pn_A$$

Partner notification also affects the total testing rate, which is now:

$$scr \; + \; S\,trt \; + \; U\,pn_U + \; A\,pn_A$$

4.2. Statistical model

Both new parameters had uninformative priors (exponential(0.001)). Other priors were as in the main model.

As for the main model, the full log-likelihood was calculated by summing the log-likelihood of each individual's testing data, multiplied by their survey weight.

- For individuals who were not tested for chlamydia in the last year, the likelihood was:
$$P_{Poisson}(0|scr + S\ trt + \ U\ pn_U + \ A\ pn_A)$$

- For individuals who were diagnosed with chlamydia in the last year in response to symptoms the likelihood was the product (i.e. the log-likelihood was the sum) of two components:
  1. The likelihood associated with testing (as opposed to not testing):
  $$1 - P_{Poisson}(0|scr + S\ trt + \ U\ pn_U + \ A\ pn_A)$$
  2. The probability that a test chosen at random from all the tests reported was prompted by symptoms:
  $$\frac{S\ trt}{scr + S\ trt + \ U\ pn_U + \ A\ pn_A}$$

- For individuals who were tested for chlamydia in the last year in response to partner notification, the likelihood was the product (i.e. the log-likelihood was the sum) of three components:
  1. The likelihood associated with testing (as opposed to not testing):
  $$1 - P_{Poisson}(0|scr + S\ trt + \ U\ pn_U + \ A\ pn_A)$$
  2. The probability that a test chosen at random from all the tests reported was prompted by partner notification:
  $$\frac{U\ pn_U + \ A\ pn_A S\ trt}{scr + S\ trt + \ U\ pn_U + \ A\ pn_A}$$
  3. The likelihood of the test result, given that it was prompted by partner notification:
  $$\frac{A\ pn_A}{U\ pn_U \ + \ A\ pn_A} \quad \text{if test positive}$$
  $$\frac{U\ pn_U}{U\ pn_U \ + \ A\ pn_A} \quad \text{if test is negative}$$

- For individuals who were tested for chlamydia in the last year but not in response to symptoms, the likelihood was the product (i.e. the log-likelihood was the sum) of three components:

  1. The likelihood associated with testing (as opposed to not testing):
  $$1 - P_{Poisson}(0|scr + S\ trt)$$
  2. The probability that a test chosen at random from all the tests reported was not prompted by symptoms or partner notification:
  $$\frac{scr}{scr + S\ trt + \ U\ pn_U + \ A\ pn_A}$$
  3. The likelihood of the test result, given that is was not in response to symptoms or partner notification:
  $$\text{prevalence} = A + S \quad \text{if test positive}$$
  $$1 - \text{prevalence} = U \quad \text{if test is negative}$$
  (Screening samples individuals randomly from the population, so the probability that a screen is positive equals the population prevalence.)

4.3. Results

eFigures 8 and 9 summarize the posterior distributions from the model with partner notification. Testing due to partner notification was ~10-fold higher in infected-asymptomatic than uninfected people, because the former are more likely to have an infected partner who notifies them. However, there were few tests due to partner notification in the Natsal-3 dataset so the posterior distributions for the partner notification rates were wide, indicating uncertain parameter values. The posterior distributions of the model's other parameters were almost identical to the posteriors in the simpler model, without partner notification represented as a distinct process. Therefore, we favor the simpler model for this data set.

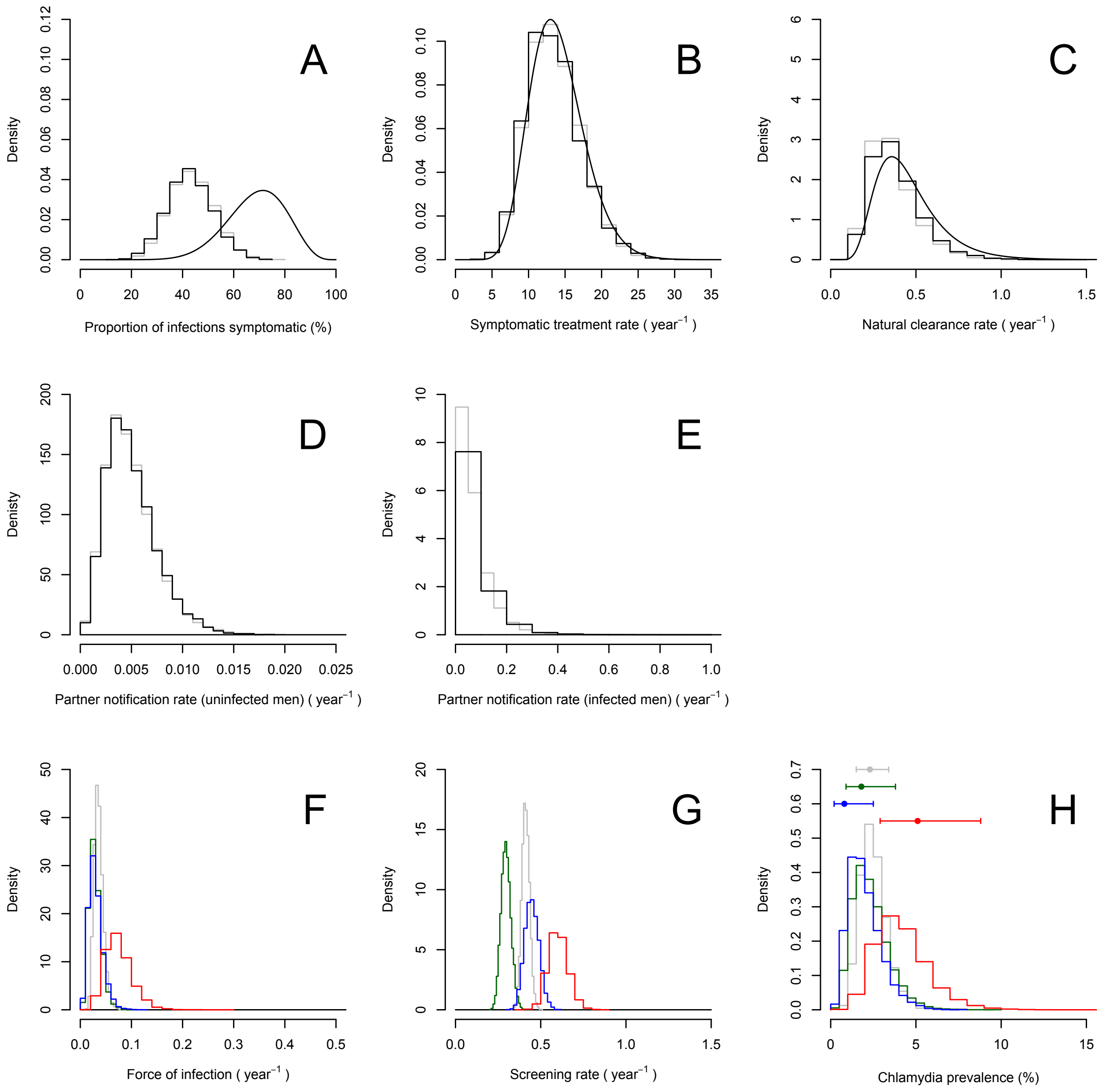

eFigure 8: Evidence synthesis to infer force of infection, screening rate and chlamydia prevalence in men aged 16-24: model with partner notification. *Panels A-G each show prior (smooth curve) and posterior (histogram) distributions for one parameter of the model. Panel H shows posterior distributions for prevalence. Gray histograms show results from analysis not stratified by number of new partners. Black or colored histograms show results from analysis stratified by number of partners: green, blue and red indicate results from individuals reporting 0, 1 or ≥2 new partners in the last year. The points and error bars in panel H indicate observed prevalence, with the 95% confidence interval.*

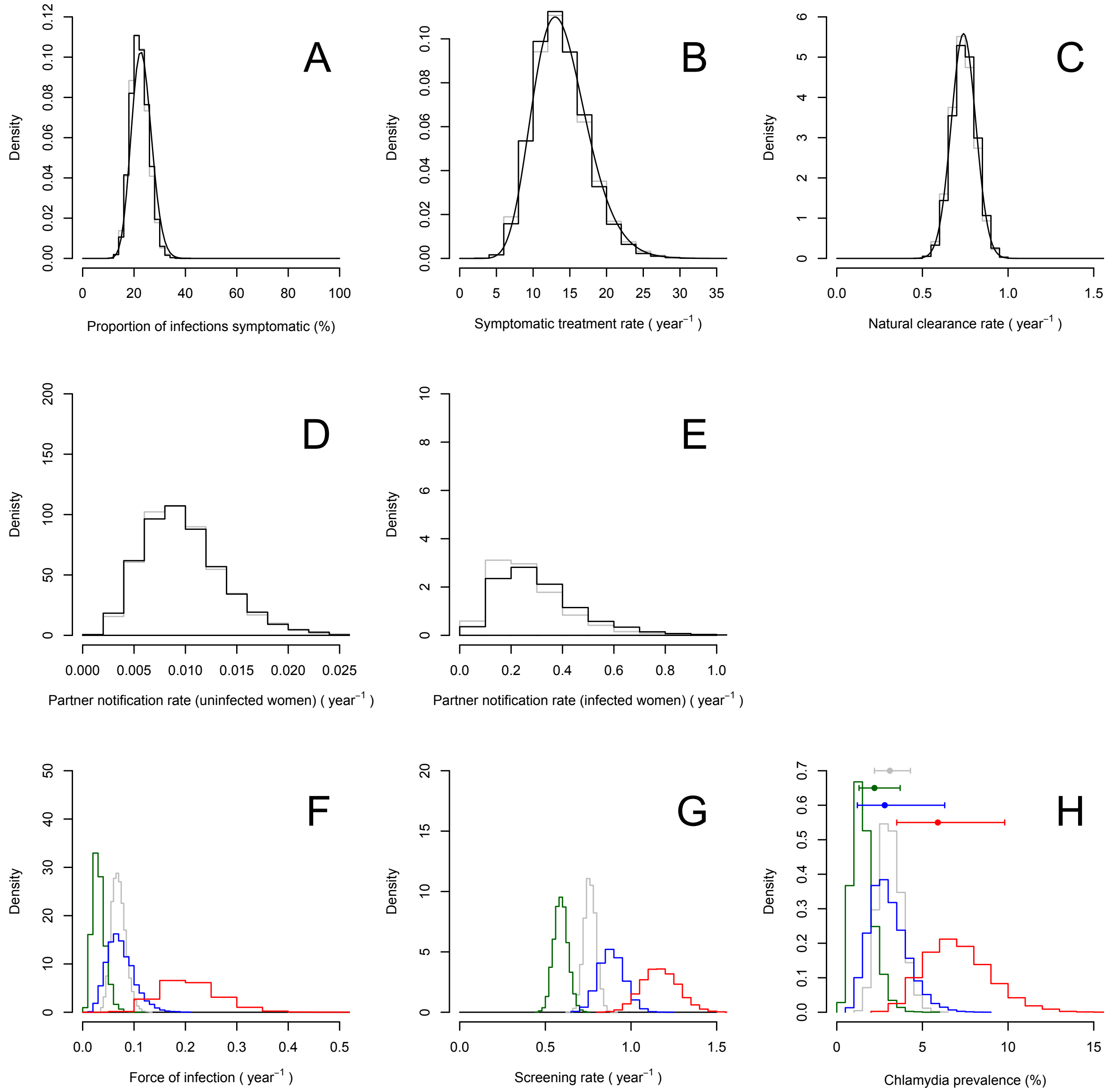

eFigure 9: Evidence synthesis to infer force of infection, screening rate and chlamydia prevalence in women aged 16-24: model with partner notification. *Panels A-G each show prior (smooth curve) and posterior (histogram) distributions for one parameter of the model. Panel H shows posterior distributions for prevalence. Gray histograms show results from analysis not stratified by number of new partners. Black or colored histograms show results from analysis stratified by number of partners: green, blue and red indicate results from individuals reporting 0, 1 or ≥2 new partners in the last year. The points and error bars in panel H indicate observed prevalence, with the 95% confidence interval.*

5. References

1. Woodhall SC, Soldan K, Sonnenberg P, et al., Is chlamydia screening and testing in Britain reaching young adults at risk of infection? Findings from the third National Survey of Sexual Attitudes and Lifestyles (Natsal-3) *Sexually Transmitted Infections.* 2016;92:218-227.
2. Geisler WM, Wang C, Morrison SG, et al., The natural history of untreated Chlamydia trachomatis infection in the interval between screening and returning for treatment. *Sexually Transmitted Diseases.* 2008;35:119-123.
3. Price MJ, Ades AE, De Angelis D, et al., Mixture-of-exponentials models to explain heterogeneity in studies of the duration of Chlamydia trachomatis infection. *Statistics in Medicine.* 2013;32:1547-1560.
4. Lewis J, Price MJ, Horner PJ, et al., Genital C. trachomatis infections clear more slowly in men than women, but are less likely to become established. *Journal of Infectious Diseases.* 2017;216:237-244.
5. Lewis J and White PJ, Estimating local chlamydia incidence and prevalence using surveillance data. *Epidemiology.* 2017;28:492-502.
6. Mercer CH, Sutcliffe L, Johnson AM, et al., How much do delayed healthcare seeking, delayed care provision, and diversion from primary care contribute to the transmission of STIs? *Sexually Transmitted Infections.* 2007;83:400-405.
7. Sonnenberg P, Clifton S, Beddows S, et al., Prevalence, risk factors, and uptake of interventions for sexually transmitted infections in Britain: findings from the National Surveys of Sexual Attitudes and Lifestyles (Natsal). *Lancet.* 2013;382:1795-1806.
